# A cognitive bias in diagnostic reasoning and its remediation by the “2-Dimensional Approach”

**DOI:** 10.15694/mep.2020.000123.1

**Published:** 2020-06-18

**Authors:** Hisashi Shimozono, Nobutoshi Nawa, Makoto Takahashi, Makoto Tomita, Yujiro Tanaka

**Affiliations:** 1 Department of Medical Education Research and Development; 2 Clinical Research Center

**Keywords:** the 2D Approach, a bias for either organs or systems, cognitive bias, clinical reasoning, diagnosis.

## Abstract

This article was migrated. The article was marked as recommended.

Background

Misdiagnoses are associated with various cognitive biases, which are difficult to reduce even if physicians think about clinical cases deliberately. We propose a new “2-Dimensional Approach” that combines two perspectives in diagnostic reasoning: organs (brain, heart, lung, intestine/liver, kidney) and systems (metabolic, endocrine, blood/tumor, infection, immune, circulatory, nervous systems). Systems involve throughout the whole body and can be pathogenesis of diseases. As a result of diseases, organs show abnormal vital signs and symptoms. We investigated: (1) whether each individual resident has a bias for either organs or systems when they diagnose clinical cases, and (2) whether the 2D Approach can reduce such a bias.

Methods

We randomly assigned PGY1 residents (n=105) to either the 2D Approach group (n=45) or a control group (n=60). After attending either a lecture on the 2D Approach or a non-related lecture, residents were asked to diagnose two clinical cases. We divided each diagnosis into one of the two categories, either “organs” or “systems”. We investigated whether each resident would diagnose the two cases into the same category, either organs or systems (i.e., a bias for either organs or systems).

Results

The participants in the control group tended to diagnose the two cases into the same category, either organs or systems (OR: 5.63, 95% CI: 1.62-21.7, p=0.0030, Fisher’s exact test). In the 2D Approach group, the category of diagnoses for the two cases were not related to each other (OR: 2.14, 95% CI: 0.50-9.81, p=0.33).

Conclusion

There is a bias for either organs or systems when residents diagnose clinical cases, suggesting that organs and systems are different perspectives in diagnostic reasoning. By combining these 2 different perspectives in a 2-dimensional matrix, the 2D Approach reduces this bias.

## Introduction

Clinical reasoning is one of the most important competence of a physician ( Norman, 2005). The dual process theory from cognitive psychology ( Stanovich and West, 2000; Evans, 2003; Kahneman, 2003) showed that the reasoning process consists of System 1 and System 2 processes ( Croskerry, 2009; Norman, 2009). System 1 is an intuitive thinking process, which is unconscious and quick, while System 2 is an analytical thinking process, which is deliberate and slow ( Kahneman, 2011).

System 1 is defined as heuristics or mental shortcuts automatically employed by expert physicians. Physicians match the pattern of clinical cases to stored illness scripts that are developed in their memory through clinical experiences ( Charlin *et al.*, 2007; Schmidt and Rikers, 2007). This intuitive process enables physicians to diagnose clinical cases quickly, but at the same time it is prone to various cognitive biases ( Croskerry, 2003b).

To reduce these biases physicians use the System 2 process that assesses whether a diagnosis made using the intuitive process is correct or not by analyzing more information ( Pelaccia *et al.*, 2011; Croskerry *et al.*, 2013). However, even the System 2 process is prone to premature closure and confirmation bias ( Stiegler and Gaba, 2015) and there is no sufficient evidence that debiasing strategies improve diagnostic accuracy ( Norman *et al.*, 2017).

On the other hand, strategies based on the reorganization of knowledge are considered to improve diagnostic accuracy ( Norman *et al.*, 2017). Previously, we developed the 2-Dimensional Approach and showed that it improves junior residents’ clinical reasoning by encouraging them to organize their knowledge ( Shimozono *et al.*, 2019). In this approach, we use two large categories, systems and organs, which cover all possible domains in internal medicine. Systems (metabolic, endocrine, blood/tumor, infection, immune, circulatory, and nervous systems) circulate around the body and become pathogenesis. Systems are almost the same as Dr. Collins’ “VINDICATE”, which is a checklist for listing differential diagnoses based on pathogeneses ( Collins, 1981). In our novel 2-Dimensional Approach, we also have organs (brain, heart, lung, liver/intestine, and kidney), which show symptoms and signs as a result of diseases. We consider that these two perspectives are the key not only for knowledge organization but also for debiasing, because they can help physicians switch their viewpoint from organs to systems, and vice versa.

We present two examples to show the difficulty in switching the viewpoint from organs to systems and vice versa, which may be alleviated by using the 2D Approach. Our first example is the differential diagnosis of hypotension. The physiology of the heart navigates physicians to preload (hypovolemia, bleeding), cardiac contractility (myocardial infarction, valvular disease, arrhythmia), afterload (dysautonomia, sepsis, anaphylaxis), and obstructive processes (pulmonary embolism, tension pneumothorax, cardiac tamponade). However, considering the physiology of the heart does not always navigate physicians to adrenal deficiency, as the switchover from the viewpoint of organs (in this case, the heart) to systems (in this case, endocrine) is difficult.

Similarly, it also may be difficult to switch the viewpoint from systems to organs. Our second example is the differential diagnoses of hypoglycemia. Considering the condition from the viewpoint of systems navigates us to endocrine disorders (insulinoma, adrenal deficiency), metabolic disorders (ketotic hypoglycemia, glycogen storage disease), and medications, but not always to liver cirrhosis, as the liver is an organ.

**Figure 1.**
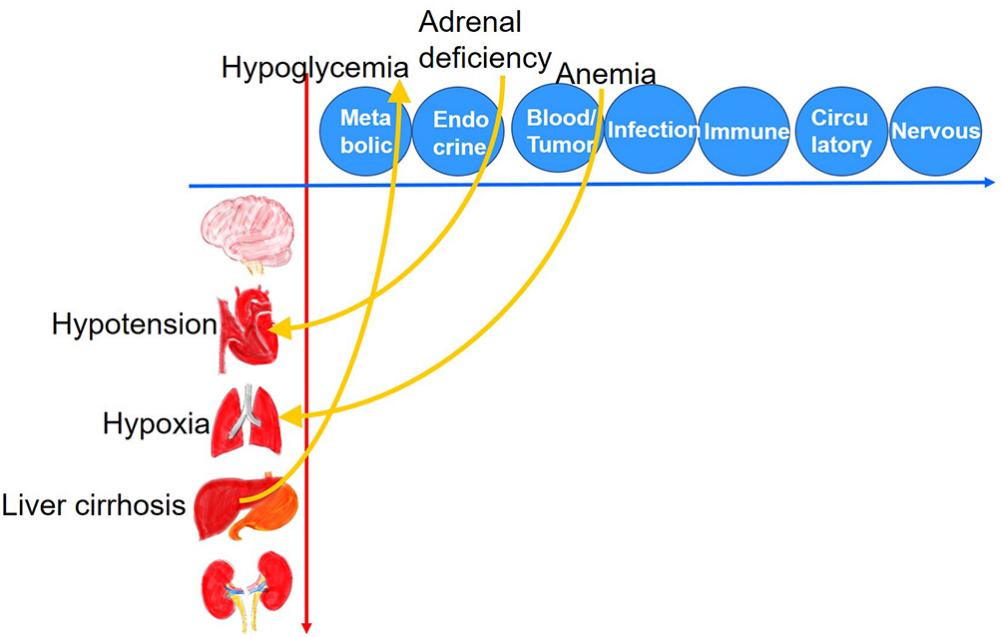
The 2-dimensional matrix visualizes the cause-and-effect relationship between organs and systems. This figure shows adrenal deficiency as a cause of hypotension and liver cirrhosis as a cause of hypoglycemia. As an example that is difficult to notice, severe anemia causes hypoxia. (modified from the figures published in ‘“The 2-Dimensional Approach”: a novel tool to help learners organize their knowledge and improve their clinical reasoning skills.’ ( Shimozono *et al.*, 2019).)

In contrast, the dual-perspective of organs and systems in the 2D Approach can help visualize cause-and-effect relationships between organs and systems on the 2-dimensional matrix ( Figure 1), which may help learners switch their viewpoint from organs to systems, and vice versa.

The study objectives were to investigate: (1) whether each individual resident has a bias for either organs or systems when they diagnose clinical cases, and, if such a bias is observed, (2) whether the 2D Approach reduces such a bias.

## Methods

### Participants

This study was a randomized control study. Participants were the 2018 first year postgraduate (PGY-1) residents of the Tokyo Medical and Dental University Medical Hospital. Among 118 residents, 113 residents attended an orientation session held as a residency program on March 30, 2018. Among them, 105 residents provided written informed consent.

This informed consent stated that their scores on the medical licensing examination would be used as data for our study. In Japan, the medical licensing examination consists of two parts, knowledge questions and clinical cases, and the scores for these two parts are disclosed by the Ministry of Health, Labour and Welfare. According to these two scores, we stratified the participants and randomly assigned them to either the 2D Approach group (n=45) or a control group (n=60) using the Hope eACReSS system (Fujitsu, Tokyo, Japan). Full marks in the knowledge questions were 200 points and the minimum standard for a pass was 160 points, so we stratified at intervals of 10 (i.e.; 160~169, 170~179, 180~189, 190~200). As full marks in the clinical cases were 300 points and the minimum standard for a pass was around 210, we stratified the results at intervals of 20 (i.e.; ~219, 220~239, 240~259, 260~279, 280~300).

### Procedure

The 2D Approach group had a one-hour lecture on the 2D Approach, and the control group had a non-related lecture about the protection of personal information. The essence of the lecture on the 2D Approach is described in the Introduction. More precise contents including an example case are shown in the Supplementary File 1.

After each lecture, the participants were asked to diagnose two clinical cases. We divided each diagnosis into one of the two categories, either organs or systems, and investigated whether each participant had a bias for either organs or systems in their diagnoses. Case 1 presented with dizziness and difficulty in walking. Neurological examination showed no abnormal findings other than walking difficulty. Blood tests revealed hyponatremia. Although the patient also presented with SIADH (system), the correct diagnosis was cerebellar infarction (organ). Case 2 presented with prolonged vomiting and diarrhea. Physical examination showed tremor and tachycardia. Blood tests revealed no abnormal findings. Physicians may misdiagnose such a case as gastroenteritis (organ); however, the correct diagnosis was hyperthyroidism (system).

After the examination, the 2D Approach group had the lecture on the protection of personal information and the control group was given the lecture on the 2D Approach in order for every resident to attend both lectures.

### Data Analysis

We used Fisher’s exact test to investigate the relationship between the category (organs or systems) of diagnoses for Case 1 and Case 2, in the 2D Approach group and the control group. We also performed subgroup analysis. As the 2D Approach had an effect on knowledge organization only among residents with adequate knowledge in our previous study ( Shimozono *et al*., 2019), we divided the participants into two groups, a high-knowledge group and a low-knowledge group, using their median score on the knowledge questions in the medical licensing examination. In both of these groups, we investigated the relationship between the category (organs or systems) of diagnoses for Case 1 and Case 2, in the 2D Approach group and the control group, using Fisher’s exact test.

We used EZR (Saitama Medical Center, Jichi Medical University, Saitama, Japan) for data analysis. EZR is a modified version of R (The R Foundation for Statistical Computing, Vienna, Austria) ( Kanda, 2013). This study has ethics approval of the Institutional Review Board in Tokyo Medical and Dental University (M2017-318).

## Results/Analysis

There was no significant difference in the scores on the medical licensing examination between the 2D Approach group and the control group (Knowledge questions: 181.9 vs. 182.0, p=0.94; Clinical cases: 244.8 vs. 241.6, p=0.17, by two-sample t test), and the two groups’ scores on the medical licensing examination were matched.

In the control group (n=58), the category of diagnoses for Case 1 and Case 2 had a significant relationship. Diagnosing Case 1 as a disease of “systems” was related to increased likelihood of diagnosing Case 2 as a disease of “systems” (OR: 5.63, 95% CI: 1.62-21.7, p=0.0030, Fisher’s exact test). In the 2D Approach group (n=43), the category of diagnoses for Case 1 and Case 2 were not related (OR: 2.14, 95% CI: 0.50-9.81, p=0.33) ( Table 1). Four participants did not diagnose Case 2 and they were excluded from the analysis.

**Table 1.** Relationship between the category (organs or systems) of diagnoses for Case 1 and Case 2. The tables show the results in (a) the control group (n=58) and (b) the 2D Approach group (n=43).

Case 1		Case 2	
organs	systems	organs	systems
24	10	7	17

OR: 5.63, 95% CI: 1.62-21.7, p=0.0030

(b) The 2D Approach group (n=43)

**Table:** 

Case 1		Case 2	
organs	systems	organs	systems
18	6	11	8

OR: 2.14, 95% CI: 0.50-9.81, p=0.33

As a subgroup analysis, we divided the participants into two groups, a high-knowledge group and a low-knowledge group, according to their scores on the knowledge questions in the medical licensing examination. We used their median score (183 out of 200) as the cut-off point. Among the high-knowledge group (n=52), the category of diagnoses for Case 1 and Case 2 were marginally associated with each other in the control group (OR: 4.66, 95% CI: 0.81-32.5, p=0.066), whereas in the 2D Approach group, the category of diagnoses for Case 1 and Case 2 were not related to each other (OR: 1.67, 95% CI: 0.10-28.1, p=1) ( Table 2). Among the low-knowledge group (n=49), the category of diagnoses for Case 1 and Case 2 were significantly related to each other in the control group (OR: 5.58, 95% CI: 0.83-48.7, p=0.048), whereas in the 2D Approach group, the category of diagnoses for Case 1 and Case 2 were not related to each other (OR: 2.16, 95% CI: 0.28-19.1, p=0.66) ( Table 3).

**Table 2.** Relationship between the category (organs or systems) of diagnoses for Case 1 and Case 2 among the high-knowledge group (n=52). The tables show the results in (a) the control group (n=29) and (b) the 2D Approach group (n=23).

Case 1		Case 2	
organs	systems	organs	systems
9	5	4	11

OR: 4.66, 95% CI: 0.81-32.5, p=0.066

(b) The 2D Approach group (n=23)

**Table:** 

Case 1		Case 2	
organs	systems	organs	systems
12	2	7	2

OR: 1.67, 95% CI: 0.10-28.1, p=1

**Table 3.** Relationship between the category (organs or systems) of diagnoses for Case 1 and Case 2 among the low-knowledge group (n=49). The tables show the results in (a) the control group (n=29) and (b) the 2D Approach group (n=20).

Case 1		Case 2	
organs	systems	organs	systems
15	5	3	6

OR: 5.58, 95% CI: 0.83-48.7, p=0.048

(b) The 2D Approach group (n=20)

**Table:** 

Case 1		Case 2	
organs	systems	organs	systems
6	4	4	6

OR: 2.16, 95% CI: 0.28-19.1, p=0.66

The diagnostic accuracy for the two cases was 16.2% and 23.8%, respectively, among all the participants. There was no significant difference between the 2D Approach group and the control group by Fisher’s exact test (Case 1: 20.0% vs. 13.3%, p=0.43; Case 2: 22.2% vs. 25.0%, p=0.82).

## Discussion

The scores of the 2D Approach group and the control group were matched for the medical licensing examination (knowledge questions, clinical cases). Case 1 and Case 2 seemed to be difficult for residents, and the participants diagnosed the cases as various diseases of organs and systems. We evaluated a bias for either organs or systems in their diagnoses. In the control group the participants tended to diagnose both cases into the same category of diseases, either organs or systems, while there was no such a bias in the 2D Approach group. Sub-group analysis detected this bias among the control group in both knowledge level groups, whereas among the 2D Approach group, we did not observe such a bias in either knowledge level group.

At first, this study revealed that residents in the control group, no matter how much knowledge they may have, tended to diagnose complicated cases into the same category of diseases, either organs or systems. We call this new cognitive bias an “organ or system bias”. Hashem *et al.* ( Hashem *et al.*, 2003) reported that specialists tend to diagnose clinical cases outside their domain as diseases within their domain. The organ or system bias in residents is a more generalized version of this bias. Through clinical experience, physicians gradually become more focused on one organ or one system, rather than organs or systems. The presence of the organ or system bias, which even novice learners have, means that two viewpoints of organs and systems are different types of scale, which the 2D Approach combines as a 2-dimensional matrix.

Secondly, it is not surprising that the 2D Approach reduced the organ or system bias, as it combines both perspectives in a 2-dimensional matrix. Its debiasing effect was not related to knowledge level, in contrast to our findings that knowledge level influenced knowledge organization ( Shimozono *et al*., 2019). Therefore, the debiasing effect of the 2D Approach is an unconscious process rather than a conscious process that requires deep knowledge ( Mamede *et al.*, 2010a). We consider that this debiasing effect is similar to Nudge ( Thaler and Sunstein, 2008), whereby a person is encouraged to change their decisions better even though they may not notice it. The dual perspective of the 2D Approach nudges and encourages subjects to move their focus from a single perspective to multiple perspectives.

There have been some strategies proposed for debiasing, such as (1) slowing down to increase reliance on System 2 ( Moulton *et al.*, 2007; Norman *et al.*, 2014), (2) cognitive forcing strategy to help learners notice their cognitive biases ( Croskerry, 2003a; Reilly *et al.*, 2013; Sherbino *et al.*, 2014), and (3) guided reflection to counteract availability bias ( Mamede *et al.*, 2010b). Among them, reflection is based on the reorganization of knowledge and improves diagnostic accuracy ( Norman *et al.*, 2017). Our previous study also reported that the 2D Approach improved junior residents’ clinical reasoning by encouraging them to organize their knowledge ( Shimozono *et al*., 2019). Considering the results on bias in this study, the 2D Approach may have the same effects as reflection, that is, an effect on bias and an effect on knowledge organization.

However, there are some differences between reflection and the 2D Approach. One difference is on bias. Reflection requires conscious effort to review differential diagnoses based on structured instructions ( Mamede *et al.*, 2010b). On the contrary, we do not need any conscious effort to switch the viewpoints between organs and systems in the 2D Approach because the 2-dimensional matrix is nudging us to do so by itself.

Another difference is on knowledge organization. The 2D Approach can help physicians visualize the pathophysiology of patients by organizing cause-and-effect relationships between organs and systems in the 2-dimensional matrix (Supplementary File 1), while reflection is a thinking process and as such, it is difficult to visualize the pathophysiological understanding.

Thus, the 2D Approach achieves a balance in clinical reasoning, nudging physicians to switch their viewpoints between organs and systems automatically, and at the same time helping them organize their knowledge deliberately based on pathophysiology.

### Limitations

Our study has several limitations. Firstly, our sample was derived from a single institution, which may limit the generalizability of the findings. Future studies with multiple institutions are warranted for external validity. Secondly, although we did not observe the effect of the 2D Approach on diagnostic accuracy, there is a possibility that two clinical cases cover too few domains and we may need to analyze more cases covering more domains.

## Conclusion

We found that junior residents tended to diagnose complex clinical cases within either the organs category or the systems category, whereas there was no such a bias in residents who had a lecture on the 2D Approach. These results suggest that there is a bias for either organs or systems when residents with limited experience diagnose clinical cases, and the 2D Approach reduces such a bias. Sub-group analysis revealed that this phenomenon is not related to residents’ knowledge level.

## Take Home Messages

• Cognitive biases are difficult to reduce even if physicians think about clinical cases deliberately.
• Each individual resident has a bias for either organs or systems when they diagnose clinical cases, suggesting that organs and systems are different perspectives in diagnostic reasoning.
• By combining the two different perspectives of organs and systems in a 2-dimensional matrix, the 2D Approach reduces this bias for either organs or systems.

## Notes On Contributors

**Hisashi Shimozono** is a PhD student, Department of Medical Education Research and Development, Graduate School of Medical and Dental Sciences, Tokyo Medical and Dental University (ORCID: https://orcid.org/0000-0001-7670-5486).

**Nobutoshi Nawa** is an Associate Professor, Department of Medical Education Research and Development, Graduate School of Medical and Dental Sciences, Tokyo Medical and Dental University.

**Makoto Takahashi** was an Associate Professor, Department of Medical Education Research and Development, Graduate School of Medical and Dental Sciences, Tokyo Medical and Dental University when this study was started, and currently he is a Professor, Center for Medical Education and International Relations, Faculty of Medicine, Hokkaido University (ORCID: https://orcid.org/0000-0001-5810-1224).

**Makoto Tomita** was an Associate Professor, Clinical Research Center, Tokyo Medical and Dental University Hospital of Medicine when this study was started, and currently he is a Professor, School of Data Science, Yokohama City University.

**Yujiro Tanaka** is a Professor, Department of Medical Education Research and Development, Graduate School of Medical and Dental Sciences, Tokyo Medical and Dental University (ORCID: https://orcid.org/0000-0002-3895-1886).

## References

[ref1] Charlin B. Boshuizen H. P. Custers E. J. and Feltovich P. J. (2007) Scripts and clinical reasoning. Med Educ. 41(12), pp.1178–1184. 10.1111/j.1365-2923.2007.02924.x 18045370

[ref2] Collins R.D. (1981) Dynamic differential diagnosis. Philadelphia: J. B. Lippincott Company.

[ref3] Croskerry P. (2003a) Cognitive forcing strategies in clinical decisionmaking. Annals of Emergency Medicine. 41(1), pp.110–120. 10.1067/mem.2003.22 12514691

[ref4] Croskerry P. (2003b) The importance of cognitive errors in diagnosis and strategies to minimize them. Academic Medicine. 78(8), pp.775–780. 10.1097/00001888-200308000-00003 12915363

[ref5] Croskerry P. (2009) A universal model of diagnostic reasoning. Acad Med. 84(8), pp.1022–1028. 10.1097/ACM.0b013e3181ace703 19638766

[ref6] Croskerry P. Singhal G. and Mamede S. (2013) Cognitive debiasing 1: origins of bias and theory of debiasing. Bmj Quality & Safety. 22, pp.ii58–ii64. 10.1136/bmjqs-2012-001712 PMC3786658 23882089

[ref7] Evans J. S. T. (2003) In two minds: dual-process accounts of reasoning. Trends in Cognitive Sciences. 7(10), pp.454–459. 10.1016/j.tics.2003.08.012 14550493

[ref8] Hashem A. Chi M. T. H. and Friedman C. P. (2003) Medical errors as a result of specialization. Journal of Biomedical Informatics. 36(1-2), pp.61–69. 10.1016/s1532-0464(03)00057-1 14552847

[ref9] Kahneman D. (2003) A perspective on judgment and choice: mapping bounded rationality. Am Psychol. 58(9), pp.697–720. 10.1037/0003-066x.58.9.697 14584987

[ref10] Kahneman D. (2011) Thinking fast and slow. Great Britain: Penguin Books.

[ref11] Kanda Y. (2013) Investigation of the freely available easy-to-use software ‘EZR’ for medical statistics. Bone Marrow Transplant. 48(3), pp.452–458. 10.1038/bmt.2012.244 23208313 PMC3590441

[ref12] Mamede S. Schmidt H. G. Rikers R. M. Custers E. J. (2010a) Conscious thought beats deliberation without attention in diagnostic decision-making: at least when you are an expert. Psychol Res. 74(6), pp.586–592. 10.1007/s00426-010-0281-8 20354726 PMC2938445

[ref13] Mamede S. van Gog T. van den Berge K. Rikers R. . (2010b) Effect of Availability Bias and Reflective Reasoning on Diagnostic Accuracy Among Internal Medicine Residents. Jama-Journal of the American Medical Association. 304(11), pp.1198–1203. 10.1001/jama.2010.1276 20841533

[ref14] Moulton C. A. E. Regehr G. Mylopoulos M. and MacRae H. M. (2007) Slowing down when you should: A new model of expert judgment. Academic Medicine. 82(10), pp.S109–S116. 10.1097/ACM.0b013e3181405a76 17895673

[ref15] Norman G. (2005) Research in clinical reasoning: past history and current trends. Med Educ. 39(4), pp.418–427. 10.1111/j.1365-2929.2005.02127.x 15813765

[ref16] Norman G. (2009) Dual processing and diagnostic errors. Adv Health Sci Educ Theory Pract. 14 Suppl 1, pp.37–49. 10.1007/s10459-009-9179-x 19669921

[ref17] Norman G. Sherbino J. Dore K. Wood T. (2014) The Etiology of Diagnostic Errors: A Controlled Trial of System 1 Versus System 2 Reasoning. Academic Medicine. 89(2), pp.277–284. 10.1097/acm.0000000000000105 24362377

[ref18] Norman G. R. Monteiro S. D. Sherbino J. Ilgen J. S. (2017) The Causes of Errors in Clinical Reasoning: Cognitive Biases, Knowledge Deficits, and Dual Process Thinking. Acad Med. 92(1), pp.23–30. 10.1097/acm.0000000000001421 27782919

[ref19] Pelaccia T. Tardif J. Triby E. and Charlin B. (2011) An analysis of clinical reasoning through a recent and comprehensive approach: the dual-process theory. Med Educ Online. 16. 10.3402/meo.v16i0.5890 PMC3060310 21430797

[ref20] Reilly J. B. Ogdie A. R. Von Feldt J. M. and Myers J. S. (2013) Teaching about how doctors think: a longitudinal curriculum in cognitive bias and diagnostic error for residents. Bmj Quality & Safety. 22(12), pp.1044–1050. 10.1136/bmjqs-2013-001987 23955466

[ref21] Schmidt H. G. and Rikers R. M. (2007) How expertise develops in medicine: knowledge encapsulation and illness script formation. Med Educ. 41(12), pp.1133–1139. 10.1111/j.1365-2923.2007.02915.x 18004989

[ref22] Sherbino J. Kulasegaram K. Howey E. and Norman G. (2014) Ineffectiveness of cognitive forcing strategies to reduce biases in diagnostic reasoning: a controlled trial. Canadian Journal of Emergency Medicine. 16(1), pp.34–40. 10.2310/8000.2013.130860 24423999

[ref23] Shimozono H. Takahashi M. Tomita M. Takada K. (2019) “The 2-Dimensional Approach”: a novel tool to help learners organize their knowledge and improve their clinical reasoning skills. MedEdPublish. 8(2),64. 10.15694/mep.2019.000134.1 PMC10712542 38089279

[ref24] Stanovich K. E. and West R. F. (2000) Individual differences in reasoning: Implications for the rationality debate? Behavioral and Brain Sciences. 23(5), pp.645–726. 10.1017/s0140525x00003435 11301544

[ref25] Stiegler M. P. and Gaba D. M. (2015) Decision-Making and Cognitive Strategies. Simulation in Healthcare-Journal of the Society for Simulation in Healthcare. 10(3), pp.133–138. 10.1097/sih.0000000000000093 26035684

[ref26] Thaler R. H. and Sunstein C.R. (2008) Nudge: Improving decisions about health, wealth and happiness. New Haven: Yale University Press.

