# Supplementary material for: A cognitive bias in diagnostic reasoning and its remediation by the “2-Dimensional Approach”
Source: MedEdPublish (2016). 2020 Jun 18;9:123. [Version 1] doi: 10.15694/mep.2020.000123.1 (PMC10702679; doi:10.15694/mep.2020.000123.1)
Supplement: Peer review history

# Reviewer report: Reviewer response for version 1

**DOI:** 10.21956/mep.20016.r30527

**Published:** 2020-07-13

**Authors:** Megan Anakin

**Affiliations:** 1 University of Otago

This review has been migrated. The reviewer awarded 3 stars out of 5 Thank you for inviting me to review this article. I read this article with a small group of colleagues who are interested in clinical reasoning and we developed our feedback to you collaboratively. We appreciated that you have developed a teaching method to help students learn clinical reasoning by focusing on reducing biased thinking about organs or systems. We have some comments about our understanding of the article for you to consider. In the introduction, we were wondering of you would consider expanding on the two examples of cognitive biases provided in the introduction, how bias for organs or systems leads to misdiagnosis, how the cognitive biases relate to 2D approach, and explain the relationship between the 2D approach and clinical practice. Please consider explaining why dividing diagnosis into organ or systems is important. In the methods section, please consider explaining how you have operationalised ‘cognitive bias’ as a concept into an assessment item involving a written diagnosis. Please consider providing further information in the descriptions of Cases 1 and 2 to help the reader understand how they were scored as either correct or incorrect diagnosis. We were wondering why a problem list might not be an acceptable answer and if there was only one correct answer then what was the rationale for it. In the results section, the term “diagnostic accuracy” is new so please consider defining it in relation to the instrument and data collected. We are wondering if the authors what alpha level the authors set for their statistical tests because it was not stated. If we assume an alpha level of 0.05, then the sub-group analysis shows a statistically significant organ/system bias among the control group in the low- knowledge level subgroup only. In the take home messages the authors may wish to specify the cognitive biases that relate to this article in point one. The authors may wish to revise point two because the statistical test allow differences between groups of individual not among specific individuals to be reported. Please consider revising the third take home message because the bias for organs or systems was not statistically significantly different for students in the high knowledge control group when compared to either 2D Approach sub-groups if the alpha level was 0.05. We look forward to reading a revised version of this article.

Reviewer Expertise:

NA

No decision status is available

# Reviewer report: Reviewer response for version 1

**DOI:** 10.21956/mep.20016.r30529

**Published:** 2020-07-08

**Authors:** Trevor Gibbs

**Affiliations:** 1 AMEE

This review has been migrated. The reviewer awarded 4 stars out of 5 An interesting paper which I enjoyed reading; I think that this paper also relates to a paper from the same authors regarding the 2D approach to clinical reasoning and diagnosis.This is a difficult area ( clinical reasoning) to investigate and research, shown by the number of papers in the area, so it is refreshing to read about how the authors approach the topic from a different standpoint. I do think that the authors put into a more constructed form what many faculty do teach, without having a solid foundation behind them- moving between systems 1 and 2 as they teach. Hence I think I would have liked a little more information on the teaching that followed after the lectures, was this standardised or dependent on individual faculty.However, I do think that this paper adds to our present knowledge on clinical reasoning and would be happy to recommend it.

Reviewer Expertise:

NA

No decision status is available

# Reviewer report: Reviewer response for version 1

**DOI:** 10.21956/mep.20016.r30528

**Published:** 2020-06-20

**Authors:** sathyanarayanan varadarajan

**Affiliations:** 1 SRM Medical College, SRM University

This review has been migrated. The reviewer awarded 4 stars out of 5 Thanks for the invite. This interesting research article is based on a randomized control study done among first year postgraduate (PGY-1) residents of Tokyo Medical and Dental University Medical Hospital, investigating a novel tool which might help learners to organize their knowledge and improve their clinical reasoning skills, known as “2-Dimensional Approach”. The authors investigated, whether each individual resident has a bias for either organs or systems when they diagnose clinical cases, and, if such a bias is observed, whether the 2D Approach reduces such a bias. The study was conducted systematically with appropriate study design & methodology and statistically analyzed suitably. The results suggest that there is a bias for either organs or systems when residents with limited experience diagnose clinical cases, and the 2D approach reduces such a bias. Though this study has several limitations like limited generalizability of the findings and covers too few domains, it’s a good contribution on the cognitive bias and the 2D Approach. It shows that by combining the two different perspectives of organs and systems in a 2-dimensional matrix, the 2D Approach reduces this bias for either organs or systems.

Reviewer Expertise:

NA

No decision status is available
